# Synthetic lethality in lung cancer and translation to clinical therapies

**DOI:** 10.1186/s12943-016-0546-y

**Published:** 2016-09-29

**Authors:** Ada W. Y. Leung, Tanya de Silva, Marcel B. Bally, William W. Lockwood

**Affiliations:** 1 Experimental Therapeutics, BC Cancer Research Centre, 675 West 10th Ave, Vancouver, BC V5Z 1L3 Canada; 2 Department of Pathology and Laboratory Medicine, University of British Columbia, Rm. G227-2211 Wesbrook Mall, Vancouver, BC V6T 2B5 Canada; 3 Integrative Oncology, BC Cancer Research Centre, 675 West 10th Ave, Vancouver, BC V5Z 1L3 Canada; 4 Faculty of Pharmaceutical Sciences, University of British Columbia, 2405 Wesbrook Mall, Vancouver, BC V6T 1Z3 Canada; 5 Centre for Drug Research and Development, 2405 Wesbrook Mall, Vancouver, BC V6T 1Z3 Canada

**Keywords:** Lung cancer, Synthetic lethality, Combination treatments, Synergy, Drug-drug interactions

## Abstract

Lung cancer is a heterogeneous disease consisting of multiple histological subtypes each driven by unique genetic alterations. Despite the development of targeted therapies that inhibit the oncogenic mutations driving a subset of lung cancer cases, there is a paucity of effective treatments for the majority of lung cancer patients and new strategies are urgently needed. In recent years, the concept of synthetic lethality has been established as an effective approach for discovering novel cancer-specific targets as well as a method to improve the efficacy of existing drugs which provide partial but insufficient benefits for patients. In this review, we discuss the concept of synthetic lethality, the various types of synthetic lethal interactions in the context of oncology and the approaches used to identify these interactions, including recent advances that have transformed the ability to discover novel synthetic lethal combinations on a global scale. Lastly, we describe the specific synthetic lethal interactions identified in lung cancer to date and explore the pharmacological challenges and considerations in translating these discoveries to the clinic.

## Background: lung cancer – a need for new treatment strategies

Lung cancer is the leading cause of cancer mortality worldwide suffering from a late stage of disease at the time of diagnosis and a lack of effective therapeutic strategies available to treat lung tumours [1]. Lung cancer is comprised of two main subtypes: Small Cell Lung Cancer (SCLC) and Non-Small Cell Lung Cancer (NSCLC), which correspond to ~20 % and ~80 % of cases, respectively [2]. Lung adenocarcinoma (LAC) is the most common type of NSCLC, responsible for ~40 % of all lung cancer cases and, unlike other subtypes, is associated with both smokers and never smokers [2, 3]. Squamous Cell Carcinoma (SqCC) is the other major NSCLC subtype and, along with SCLC, is characterized by its development in the central airways and close association with smoking [2]. The different lung cancer subtypes develop from unique cells of origin, involve the deregulation of specific oncogenic pathways and have diverse responses to conventional chemotherapies, demonstrating the importance of considering histology in the clinical management of this disease [4].

Recently, large-scale genomics studies have revealed the genetic changes driving the development of lung cancer subtypes. Activating mutations in *EGFR* and *KRAS* as well as translocations involving *ALK* and *RET* are common in LAC while SqCC contain frequent mutations in *PIK3CA* and amplification of *FGFR1* [5]. Meanwhile, SCLCs are characterized by the dual inactivation of the tumour suppressor genes *RB* and *TP53* and, less frequently *PTEN* [6]. With this increasing understanding of lung cancer biology has come the advent of targeted therapies to combat this devastating disease. These therapies target mutated components of key cellular pathways on which tumours cells have become dependent on for survival, a phenomena known as oncogene addiction [7]. For example, tyrosine kinase inhibitors (TKIs) targeting LACs driven by mutant *EGFR* or *ALK* rearrangements have been clinically successful, highlighting the potential of designing drugs to specifically target the molecular mechanisms driving cancer development, a concept often described as “personalized medicine” [7–9]. However, despite these encouraging developments, significant problems remain. First, the majority of LAC patients are not candidates for these therapies as they have tumours without mutations in targetable genes, owing either to the lack of an identified driver or mutation in drivers such as mutant *KRAS* for which the development of inhibitors have proven elusive. Second, all patients eventually develop resistance to treatment with these targeted agents, either through secondary mutation of the target gene or downstream activation of their signalling pathways that sustain tumour growth. Furthermore, although targeted therapies have been successfully employed in the treatment of LAC, advances have lagged in SCLC and SqCC. In SCLC, the causative genetic changes involve inactivation of tumour suppressor genes - which are notoriously difficult to exploit therapeutically - while in SqCC, FGFR1 inhibitors have demonstrated mixed success, likely due to additional genetic determinants regulating response or the presence of alterative oncogene targets attributed to the amplified chromosome region [10]. In addition to the traditional targeted therapies described above, the recent efficacy and approval of inhibitors targeting the PD-1 (Programmed T cell death 1)/PD-L1 immune checkpoint in subsets of NSCLC patients has highlighted the promise of using immunotherapeutics for lung cancer treatment. However, as with kinase inhibitors, many patients do not respond to these treatments and those that do often develop resistance and efforts are already being made to understand the mechanisms regulating sustained response [11]. Thus, while undoubtedly a major advancement in improving lung cancer patient outcomes, current therapeutic approaches have failed to achieve the major goal of increasing long-term survival rates and new strategies to treat lung cancers –perhaps combining kinase inhibitors and immunotherapies - are urgently needed.

## Main text

### The concept of synthetic lethality and approaches for uncovering interactions in cancer cells

**Fig. 1:**
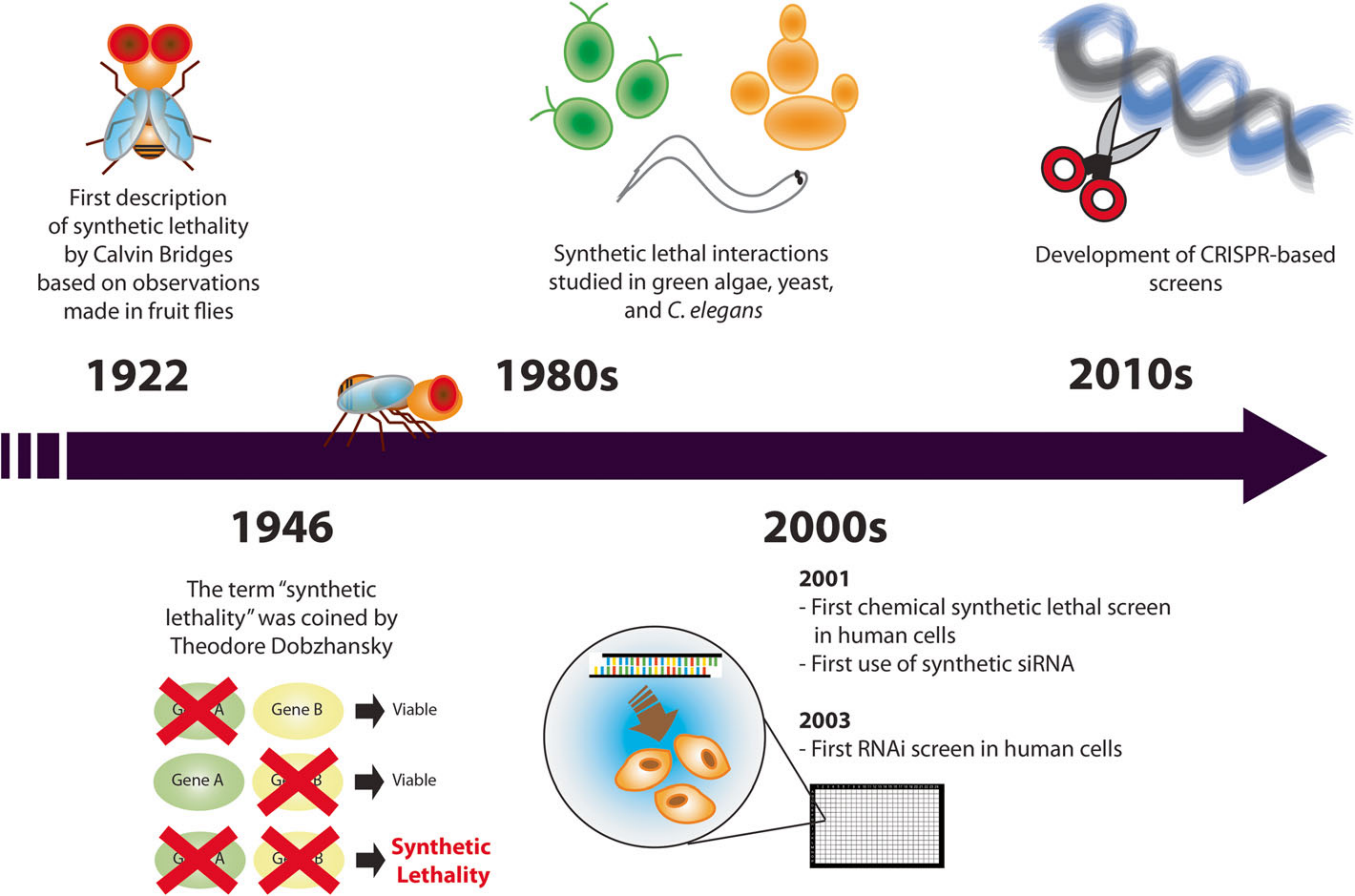
Synthetic Lethality: History and Evolution. The timeline indicates the major events that took place over the last century, from the first description of synthetic lethality to the recent development of technologies for high-throughput discoveries of synthetic lethal interactions

Synthetic lethality is traditionally defined as a condition where simultaneous mutation in two genes – but not either alone - leads to cell death [12, 13]. Where mutation in both genes impairs cellular fitness but does not cause lethality, this is described as a synthetic sick interaction [12–14]. Calvin Bridges first described synthetic lethality in 1922 when he observed that combinations of mutations in fruit flies lead to lethality (Fig. 1) [14–16]. The term itself was later coined by Theodore Dobzhansky who made the same observations in 1946 [17]. For decades, synthetic lethal interactions were studied mainly in fruit flies; however, starting in the 1980s, the search for synthetic lethal interactions expanded to other model systems including algae, yeast, and the nematode *C. elegans* [18–21]. These studies contributed significantly to our understanding of gene function, biological pathways, and genetic robustness, and identified many interactions potentially important in human cancer.

The initial concept of screening for drugs that can specifically kill cancer cells carrying defined genetic changes was originally conceived using yeast as a model system. This basic approach combined with improvements in screening platforms subsequently allowed chemical compound libraries to be assessed in human cancer cell lines, the first foray into exploring synthetic lethal targets for cancer causing genetic alterations [22]. However, chemical libraries suffer from difficulties in target identification, especially for larger libraries of diverse compounds, limiting their effectiveness in defining new synthetic lethal interactions on a global scale. Hypothesis-based assessment can alleviate these problems, as demonstrated by the successful validation of BRCA-deficiency and PARP inhibitor sensitivity in breast cancer [23]. However, the advent and rapid development of RNAi technology in the early 2000s [18, 19] allowed the first high-throughput genetic screens to be performed in human cancer cells driven by specific oncogenic mutations [24–26]. Consisting of well-based screens using transfection of individual siRNAs or pooled drop-out/enrichment screens employing transduction of lentiviral shRNA libraries, this approach has proven invaluable for identifying synthetic lethal interactions for various oncogenes/tumour suppressor genes as well as chemosensitizing genes in lung and other cancers. Recently, major advancements in the generation of RNAi libraries, sequencing, high throughput screening platforms, and the recent development of CRISPR technology have further expanded the capacity to uncover synthetic lethal interactions in cancer [27–30]. In addition, contemporary screens are now relying more on computational and bioinformatics approaches such as statistically inferring synthetic lethal interaction pairs from cancer genomic data [119]. These synthetic lethal screening approaches have been exhaustively reviewed recently [31–33].

While synthetic lethality can occur at the cellular or organismal level depending on the model being used, the goal in cancer treatment is to specifically eliminate a population of malignant cells, which is heterogeneous in nature [34]. With that perspective, drug combinations are designed with the ultimate goal of curing the disease, but more frequently, significant improvements in treatment outcomes are achieved through synergy, which we argue is equivalent to a synthetic sick effect at the cell population level in the context of cancer biology. Hence, the concept of synthetic lethality encompasses a wide range of interactions involving genetic variations as a result of single-gene mutations, chromosomal translocation and deletions, as well as cellular responses to different cytotoxic and targeting pharmaceuticals. In this review, our discussion begins with the various types of synthetic lethal interactions uncovered in the context of lung cancer. We then discuss consideration for the application and translation of these findings to treating lung cancer patients, an area that has proven challenging for the development of therapeutics based on synthetic lethal interactions to date and yet to be directly addressed in the literature.

### Types of synthetic lethality and identified interactions in lung cancer

**Fig. 2:**
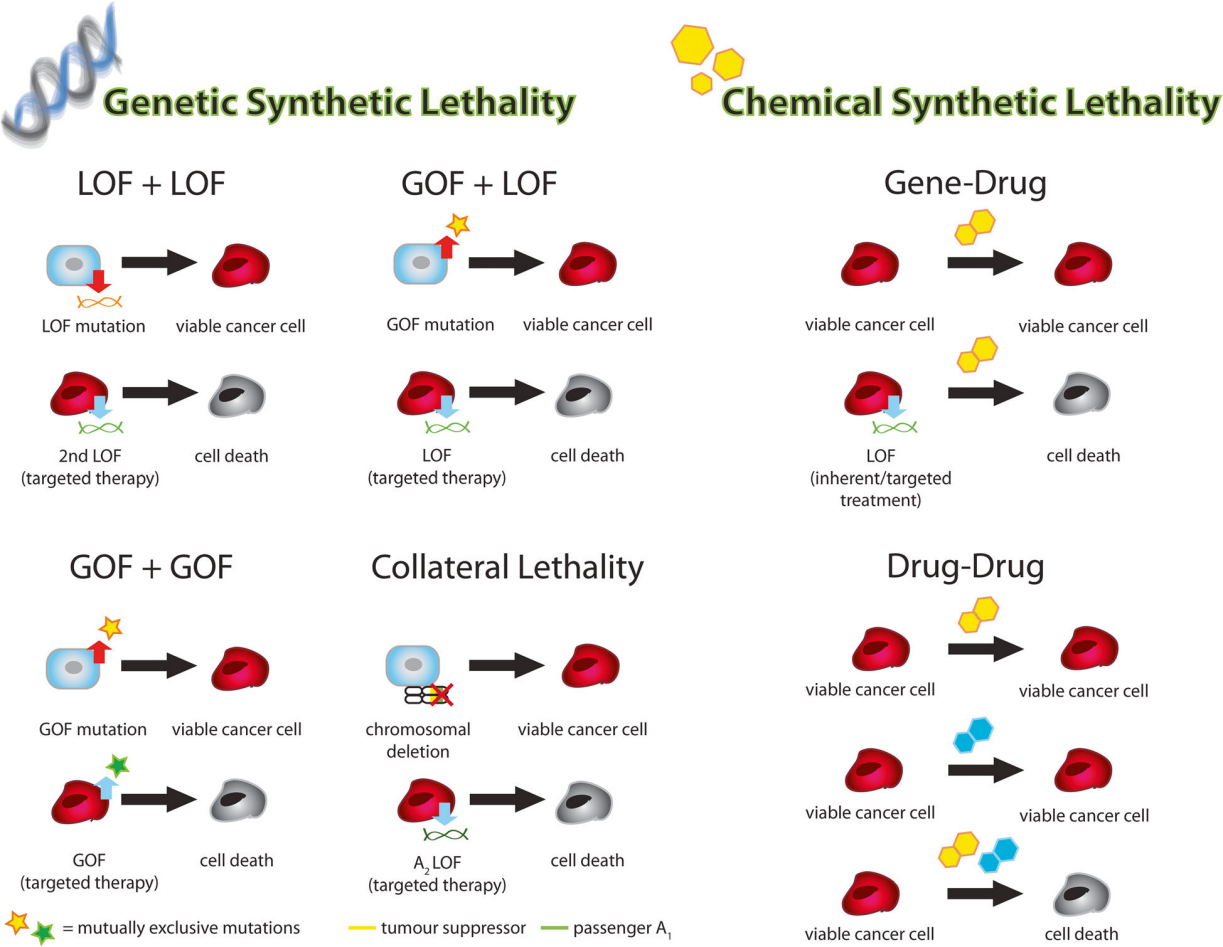
Types of Synthetic Lethal Interactions in the Context of Cancer. The various types of synthetic lethal interactions can be grouped into two categories: genetic-based and chemical-based. Genetic synthetic lethality is primarily based on cancer-specific genetic alterations (blue normal cells undergo genetic changes that result in transformation to red cancer cells) that become susceptible to further induced changes in gene expression resulting in synthetic lethality. Chemical synthetic lethality describes synthetic lethal interactions between inherent or induced genetic alterations and broad-spectrum therapeutics (chemosensitization) as well as synergistic outcomes from the use of two or more chemotherapeutics. Please see text for full description of each type of interaction. (LOF = loss-of-function, GOF = gain-of-function, passenger A_1_ = passenger gene deletion﻿, A_2﻿_ = isoform of deleted passenge﻿r A_1﻿_, blue cell = normal cell, red cell = cancer cell, grey cell = dead cancer cell)

The transformation of normal cells to cancer cells involves a step-wise evolution: a progressive series of genetic mutations that allow cells to acquire the hallmarks of cancer over time and become malignant [35]. These genetic changes cause deficiencies in, or addiction to, certain cellular processes and biological pathways that initiate transformation and are thus, prime targets for therapeutic intervention. However, cancer cells often develop resistance to broad spectrum and targeted treatments. This may first arise through cytoprotective responses and the presence of redundant (“back-up”) proteins and pathways. However, resistance will eventually arise through selection of clones that exhibit at least one mechanism required for inhibition of the drug’s action. This genetic robustness is another factor that can be targeted when using synthetic lethal strategies [13]. Here, we describe the different types of synthetic lethal interactions in two broad categories: 1) interactions that are purely based on genetic mutations and, 2) interactions that involve existing cytotoxic agents that are known to be effective in cancer patients (Fig. 2). Furthermore, we highlight specific examples of synthetic lethality that have been identified to date in lung cancer. We believe these will provide new strategies for therapeutic intervention (Table 1).

**Table 1:** Synthetic Lethal Interactions Identified in Lung Cancer

Interactor 1	Interactor 2	Type of Interaction	Method of Discovery	Lung Cancer Subtype	Year discovered	First Author	PMID
PAPSS1	Cisplatin (or other DNA damaging agents)	Chemo-sensitization	RNAi Screen (siRNA) + Low-Dose Cisplatin	LAC	2015	Leung	26220590
CABYR	Cisplatin	Chemo-sensitization	RNAi Screen (siRNA) + Cisplatin	LAC	2014	Qian Leung	24362251 26938915
dUTPase	FUdR/pemetrexed	Chemo-sensitization	Hypothesis Based	LAC; bronchioalveolar carcinoma; LCC	2012	Wilson	22172489
mKRAS	PKCi Aggegation (via Oncrasin-1 treatment)	GOF + GOF	Chemical library Compound Screen	LAC	2008	Guo	18794128
mKRAS	mEGFR	GOF + GOF	Computational - Mutual Exclusivity Analysis in Lung Cancer Genomic Data	LAC	2015	Unni/Lockwood	26047463
mKRAS	mBRAF	GOF + GOF	Hypothesis Based	LAC	2016	Cisowski	26028035
mEGFR	ARHG5	GOF + LOF	Computational - EGFR Interactome Mapping	LAC	2013	Li	24189400
mKRAS	GATA2	GOF + LOF	RNAi Screen (siRNA)	LAC	2012	Kumar	22541434
mKRAS	STK33	GOF + LOF	RNAi Screen (shRNA)	LAC	2009	Scholl	19490892
mKRAS	TBK1	GOF + LOF	RNAi Screen (shRNA)	LAC	2009	Barbie	19847166
mKRAS	PLK1	GOF + LOF	RNAi Screen (shRNA)	LAC	2009	Luo	19490893
mKRAS	WT1	GOF + LOF	RNAi Screen (shRNA)	LAC	2010	Vicent	20972333
mKRAS	CDK4	GOF + LOF	RNAi Screen (shRNA)	LAC	2010	Puyol	20609353
MYC	PRKDC	GOF + LOF	RNAi Screen (shRNA)	SCLC	2014	Zhou	25495526
mEGFR	PRKCSH + EGFR-i	GOF + LOF + LOF	RNAi Screen (shRNA) + Gefitinib	LAC	2014	Sudo	25528770
mEGFR	NF-kB + EGFR-i	GOF + LOF + LOF	RNAi Screen (shRNA/siRNA) + EGFR TKI	LAC	2011;2015	Bivona Blakely	21430781 25843712
mKRAS	BCL-XL + MEK-i	GOF + LOF + LOF	RNAi Screen (shRNA) + Selumetinib	LAC	2013	Corcoran	23245996
ATM	DNAPK	LOF + LOF	Hypothesis Based	LAC	2013	Riabinska	23761041
ATM/p53	ATR (under DNA damaging conditions)	LOF + LOF	Hypothesis Based	LAC	2011	Reaper	21490603
EGFR	Tankyrase 1	LOF + LOF	RNAi Screen (shRNA) + Gefitinib	LAC	2012	Casas-Selves	22738915
MAX	BRG1	LOF + LOF	Computational - Global gene expression analysis; cancer databases	SCLC	2014	Romero	24362264
RB1	CDKN2A	LOF + LOF	Computational - Proteome/transcriptome profiling	LAC; SCLC	2016	Kim	26647789
LKB1	phenformin	LOF + LOF	Compound library screen	LAC	2013	Shackelford	23352126
BRM/SMARCA2	BRG1	LOF + LOF/collateral	RNAi Screen (shRNA)	LAC	2014	Hoffman Orvis Wilson Oike	24520176 25115300 24421395 23872584
PSMA1 (proteosome subunit)	Radiation	Radio-sensitization	RNAi Screen (shRNA)	LAC; LCC	2013	Cron	24040035
Cisplatin	Irinotecan	Synergistic Interaction	Hypothesis Based	SCLC	2002	Noda	11784874

*GOF* gain-of-function, *LOF* loss-of-function, *LAC* lung adenocarincoma, *SCLC* small-cell lung cancer, *LCC* large cell carcinoma, *m-(gene)* mutant variant of the gene, *(gene)-i* inhibitor of gene product

### Genetic-based synthetic lethality

As mentioned above, tumour cells acquire mutations that allow them to grow rapidly over time. While these characteristics give cancerous cells advantages in proliferation and survival, they also become therapeutic targets. The differential regulation of genes between cancer cells and their corresponding normal cells allow researchers to identify those that can be targeted to induce synthetic lethality in a cancer-specific manner. The various types of interactions are described below.

#### Loss-of-function/Loss-of function

Loss-of-function (LOF) mutations in tumour suppressors are extremely common in human cancers. One such example is the genome guardian *p53*, the most frequently mutated gene in human cancers, ranging from 25 to 50 % in various tumour types including ovarian, breast, colorectal, head and neck, and lung cancers [36]. Although p53 mutations have been studied extensively, it has been a difficult target as it has no enzymatic activity and primarily functions through protein-protein interactions [37]. One approach to target this “undruggable” target has focused on strategies to restore the wild-type function of mutated p53 [38]. Another therapeutic approach exploits the vulnerabilities or genetic dependencies arising from loss of wild-type p53 functions [39]. For example, based on differential gene expression analyses, Wang and Simon proposed a list of 98 candidate genes that, when suppressed, may induce synthetic lethality in p53-deficient cancers [40]. We define such a strategy as LOF/LOF as it involves inhibiting a gene/pathway/function in the background of genetic or pharmacological inactivation of another gene to induce synthetic lethality.

To date, the biggest clinical success in synthetic lethal targeting is the use of PARP (poly (ADP-ribose) polymerase) inhibitors against tumours with BRCA mutations. BRCA1 and BRCA2 deficiencies cause defects in homologous recombination (HR) and have a significant role in hereditary breast and ovarian cancers [41]. It is known that mice lacking PARP expression are viable but are defective in repairing single-stranded DNA breaks. Loss of PARP function results in the dependence on the use of homologous recombination (HR) to repair DNA damage [42]. Based on these observations, Bryant et al. hypothesized and then demonstrated that BRCA-deficient cancers are hypersensitive to PARP inhibitors [42]. The discovery of this truly synthetic lethal interaction (by definition) has revolutionized our approach to the treatment of BRCA-associated cancers and served as the paradigm for uncovering other synthetic lethal interactions.

*PTEN*, the second most frequently mutated tumour suppressor in cancer, is a similar therapeutic target [43]. Inspired by the synthetic lethality induced by loss of BRCA2 and PARP functions, Mendes-Pereira et al. demonstrated that cancers with PTEN deficiency, which also cause HR defects, are sensitive to PARP inhibitors [44]. This synthetic lethal interaction was validated in vitro and in vivo. As a result, PARP inhibitors are currently in Phase II clinical trials for treatment of PTEN-deficient cancers. Exploiting deficiencies in DNA repair proteins has since revealed additional synthetic lethal interactions with particular relevance to lung cancer. Similar to the case of PARP and BRCA, Riabinska *et. al.* observed that cancers with ATM-deficiency are defective in HR and dependent on nonhomologous end joining (NHEJ) for DNA repair and cell survival [45]. Through genetic and pharmacological methods, they subsequently demonstrated that inhibition of the catalytic subunit of an essential NHEJ protein, DNA-dependent protein kinase (PRKDC), induces synthetic lethality specifically in ATM-deficient cancers, but not normal cells or cancer cells with active ATM. Likewise, after developing a potent and selective inhibitor of the DNA damage response (DDR) kinase ATR, Reaper and colleagues found that it induced synthetic lethality specifically in ATM- or p53-deficient cancers in the context of genotoxic stress. Since ATM is mutated and inactivated in ~10 % of LACs, these findings provide a potential therapeutic strategy for a large subset of patients, one which is especially resistant to standard chemotherapeutics.

While the above examples were mainly identified in other cancer types, many of the described genes are also disrupted in lung cancer suggesting that similar synthetic lethal interactions may also exist in this context. However, screens for lung cancer specific LOF/LOF synthetic lethal interactions have also been performed. For example, through a chemical library screen, Shackelford and colleagues found that LKB1 (STK11) deficient lung cancer cell lines were acutely sensitive to phenformin, an inhibitor of mitochondrial function [46]. LKB1 is mutated in ~20 % of NSCLC and this work, subsequently validated in Lkb1 mutant mouse models, suggests that metabolism based therapeutics may be effective in this subset of cancer patients. Using a loss-of-function whole-genome shRNA screen, inhibition of the canonical Wnt pathway, specifically the positive regulators tankyrase 1 and 2, was found to induce cell death in LAC cells only in the context of EGFR inhibition [47]. Furthermore, computational approaches have revealed LOF/LOF synthetic lethality of MAX and BRG1 in SCLC and RB1 and CDKN2A in both SCLC and NSCLC [48, 49].

Lastly, it is important to note a unique subcategory of LOF/LOF interactions that only occur in the presence of a gain-of-function (GOF) oncogene mutation. These interactions typically involve inhibiting the mutant oncogene with a small molecule inhibitor and another gene with pharmacological or genetic methods. Since the inhibitors only work – and hence synthetic lethality is only induced - in the context of the GOF mutation, we term this association GOF/LOF/LOF, even though only two genes are involved. Examples of this association include the use of EGFR kinase inhibitors in the background of mutant EGFR (mEGFR). For instance, using RNAi screening in the presence of the EGFR kinase inhibitor Erlotinib, Bivona and colleagues found that inhibiting FAS and other components of the NF-kB pathway specifically enhanced cell death in mEGFR LAC cell lines [50, 51]. Likewise, Sudo et. al. found a similar association between NF-kB inhibition and Gefitinib (another EGFR kinase inhibitor) and also identified a novel gene candidate, PRKCSH, that induced cell death in Gefitnib-treated mEGFR LAC cells [52]. Finally, a pooled shRNA screen in KRAS mutant cancer cells, which are dependent on MEK activation, found that inhibition of BCL-XL cooperated with MEK inhibitors to induce cell death [53]. These findings were validated using the MEK inhibitor selumetinib and the BCL-XL inhibitor ABT-263 in a genetically engineered KRAS-driven mouse model of lung cancer; highlighting the potential clinical relevance of this interaction.

#### Gain-of-function/Loss-of function (synthetic dosage lethality)

While loss of tumour suppressor functions is a common characteristic in cancer cells, activation of various oncogenes through gain-of-function (GOF) mutations is another major contributor to tumour development. Like LOF mutations, GOF mutations rewire cancer cells, making them susceptible to additional changes in cellular functions that would cause little or no harm to normal cells. When lethality occurs as a result of one gene being genetically activated (GOF) and another being inactivated through genetics or drug targeting (LOF), the interaction is known as synthetic dosage lethality.

As an example, constitutive activation of RAS signaling, particularly KRAS, is known to be the oncogenic driver for approximately 20 % of all human cancers [54]. Cancers of the pancreas, colon, and lung are known to have high frequencies of KRAS mutations [55]. KRAS has long been considered an “undruggable” target due to the lack of suitable binding pockets for small molecule inhibitors [56]. To target KRAS mutants, several groups have utilized high throughput RNAi screening to identify synthetic lethal partners of activated mutant KRAS. Luo et al. performed a genome-wide shRNA screen in colorectal cancer cells and found that KRAS mutants are hypersensitive to PLK1, APC/C, and proteasome inhibition relative to isogenic cells with wild-type KRAS [57]. Other synthetic lethal partners that have been identified through RNAi screening within an activated KRAS background include STK33, TBK1, WT1, CDK1, CDK4, GATA2, and Snail2, studies conducted primarily using colon and lung cancer cells [58–64]. These interactions have been reviewed elsewhere.

Hyperactivation of members of the MYC protein family is very common in a wide range of cancers. *c-MYC* is amplified in about 10 % of LAC and c-MYC, MYCL or MYCN amplification/overexpression occurs in >20 % of SCLC. MYCs are transcription factors - and thus, difficult to inhibit directly – thus, synthetic lethal strategies are being used to define novel treatments for cancers driven by the activation of these proteins. Inhibition of mTOR, Aurora A/B, SAE2, or CDK1 has been shown to induce synthetic lethality in MYC-driven cancers [65–68]. Synthetic lethal relationships between hyperactivated MYCN and suppression of BRD4 and CSNK1e have also been demonstrated in acute myeloid leukaemia and neuroblastoma, respectively [69, 70]. Importantly these interactions are being tested clinically where Phase II clinical trials are assessing therapeutic benefit of inhibitors targeting CDK1 or BRD4 in patients with MYC-amplified tumours. Recently, through a pooled shRNA screen, inhibition PRKDC was found to specifically induce synthetic lethality in SCLC cell lines with amplification/overexpression of MYC family members but not in those without [71]. This was attributed to the role of PRKDC in controlling MYC protein levels as well as facilitating repair of MYC-induced DNA damage. These results could be recapitulated with the PRKDC inhibitor, NU-7441, suggesting a possible therapeutic avenue for this aggressive lung cancer subtype.

Lastly, although EGFR kinase inhibitors have proven effective at improving outcomes of LAC patients with activating EGFR mutations, all patients treated with these inhibitors eventually develop resistance and relapse. Identifying the key factors necessary for mEGFR-mediated tumorigenesis may offer avenues for combination based therapies that can overcome EGFR kinase inhibitor resistance. Through charactering the LAC‐specific mEGFR interactome through global analysis of protein–protein interactions and phosphorylation, Li and collegues identified 8 key proteins necessary for survival mEGFR lung cancer cell lines in addition to EGFR itself: GRB2, MK12, SHC1, ARAF, CD11B, ARHG5, GLU2B, and CD11A [72]. When inhibited through siRNAs, these genes induced cell death specifically in mEGFR, but not EGFR wild-type, LAC cells. This clearly suggested a synthetic lethal relationship. Indeed, ablation of ARHG5 in mEGFR LAC cells induced apoptosis and was shown to interact with downstream EGFR signalling proteins including SHC1 and GRB2, highlighting its potential as a target for combination based therapy.

#### Gain-of-function/Gain-of-function

With the tools currently available, it is relatively easy to identify synthetic lethal interactions using gene knockdown or knockout approaches. In contrast, there has yet to be a large-scale GOF screen in human cells to search for synthetic lethality in cancers driven by known oncogenes. It is, however, not impossible to define synthetic lethal interactions that are based on two gain-of-function mutations using information derived from studies assessing human tumour evolution. For instance, it has been known for years that oncogenic mutations in KRAS and EGFR are not only common, but also mutually exclusive in LAC [73–75]. It was typically assumed that since the two oncogenes work through the activation of similar pathways, mutation in both genes was functionally redundant and thus, not positively selected during tumour development. However, in a recent study, Unni et al. discovered that these two mutations are mutually exclusive because activation of both genes in lung cells induces synthetic lethality. This explained why there is selectedion against cells expressing both mutations together during tumour evolution [76]. Expressing both mutant oncogenes in a mouse model of lung cancer led to the selection of tumours expressing a single oncogene while forced expression of mEGFR or mKRAS in LAC cells with endogenous mutations in the reciprocal oncogene induced cell death through uncontrolled macropinocytosis and catastrophic cell vacuolization. The later events were likely controlled through increased MAPK signalling. From a clinical perspective, while it is challenging to increase expression of a gene product, this study suggests that there may be opportunities to activate combinations of pathways to induce lethality. The method through which this interaction was revealed and validated suggests that additional synthetic lethal interactions may be identified through exploring co-activation of mutually exclusive driver mutations and their respective pathways. Indeed, a subsequent study revealed a similar association between mutant BRAF and mKRAS in LAC, demonstrating the validity of this approach.

Although difficult to categorize, chemical compound screens have also revealed interactions suggestive of synthetic lethality induced by GOF/GOF associations. As an example, Guo et al. identified a small molecular weight compound (oncrasin-1) that selectively and effectively killed human lung cancer cells with mKRAS through induction of apoptosis [77]. A search for a target of this novel compound revealed no changes to the activity of known RAS signalling pathway components. However, treatment with oncrasin-1 led to abnormal aggregation of PKCι in the nucleus of sensitive, but not in resistant cells. While oncrasin-1 did not change the activity of PKCι, it did induce a GOF change in terms of modifying PKCι subcellular localization. This was, in turn, associated with cell death in KRAS mutant cells. It is conceivable that compounds that stimulate or modify pathway signalling/function may also provide an opportunity to induce synthetic lethality in cancer cells in the context of GOF/GOF interactions.

#### Collateral lethality

A more recent concept of synthetic lethality has been termed “collateral lethality” [78]. In the process of malignant transformation, some tumour suppressor genes may be inactivated through chromosomal deletions. Loss of heterozygosity or homozygous deletions results in complete inactivation of a tumour suppressor; which in itself enables cancer-specific targeting of synthetic lethal partners as described above. However, collateral lethality takes advantage of “passenger” or “neighboring” genes that are co-deleted “unintentionally” in this process [78, 79]. These passenger genes may encode for housekeeping functions that are essential to cell viability but are masked by the presence of redundant genes encoded elsewhere to complement for the loss. Targeting homologues of these passengers forms the basis of collateral lethality. As an example, *ENO1* is a gene that is homozygously deleted in glioblastoma (GBM) as a result of deletion in the 1p36 tumour-suppressor locus [79]. This gene encodes for enolase, an enzyme that is essential for glycolysis. Although ENO1 accounts for up to 90 % of the enolase activity in GBM, its loss is tolerated through the expression of *ENO2* which is exclusively expressed in neural cells [55]. Loss of *ENO2* does not cause reduced viability in the presence of *ENO1* expression, but GBM cells with deletion in 1p36 are highly sensitive to ENO2 inhibition due to collateral lethality [79]. Muller et al. have identified other homozygously deleted essential genes that have potential collateral lethal partners in GBM [79].

To date, the only described instance of collateral lethality in lung cancer involves SMARCA4 (a chromatin remodelling helicase) and its paralogue, SMARCA2. Unlike the ENO1/ENO2 example, SMARCA4 is a true tumour suppressor gene and is directly inactivated through mutations or deletions in approximately 10–15 % of LACs. However, unlike other tumour suppressors, SMARCA4 and SMARCA2 perform cellular housekeeping functions rather than preventing abnormal cell growth. Thus, inhibition of SMARCA2 in the background of SMARCA4 genetic inactivation leads to synthetic lethality as the cell loses the ability to complete an essential function necessary for survival, as is the case with ENO1/ENO2 in GBM. Inhibitors targeting SMARCA2 may therefore prove effective in the large subset of LAC patients with SMARCA4 mutations [80–83].

### Synthetic lethal interactions involving broad-spectrum pharmaceuticals

As introduced earlier, the traditional definition of synthetic lethality/sickness involves defects (hyperactivation or inactivation) in a pair of genes. In the context of cancer, however, synthetic lethal/sick interactions also apply to gene-drug and drug-drug interactions. To date, cancer treatments rely heavily on the use of broad-spectrum therapeutics that target essential cellular processes such as DNA replication and mitosis; processes that are particularly important to rapidly dividing cells. Although these cytotoxic agents are highly effective, their uses have been limited by narrow therapeutic windows. Effective doses can result in severe and potentially life-threatening toxicities. Further, cancer cells have a remarkable capability to develop resistance against these agents over time. Nonetheless, these agents have provided significant therapeutic benefits to patients and their use makes sense even today given the nature of intra- and inter-tumoural heterogeneity. It will not be easy to supplant these agents used as standard of care for many cancers. With a better understanding of cancer genetics and the mechanisms of drug action, a tremendous amount of effort is being placed on improving the efficacy of existing cytotoxic agents through synthetic lethal approaches. From our perspective this may involve selecting the proper drug based on tumour-specific defects (chemosensitization) or developing drug combinations to achieve synergy (synthetic sickness).

#### Gene-drug interactions

Cisplatin is undoubtedly one of the most successful chemotherapeutics ever discovered for cancer treatments. While its use and clinical success is widespread, it causes severe side-effects including nephrotoxicity, neurotoxicity, and ototoxicity [84]. Furthermore, resistance to platinum based drugs is common. To overcome these challenges, many groups have attempted to develop combination products comprising cisplatin and a targeted treatment. The later can be uncovered by performing high-throughput RNAi synthetic lethal screens to better understand the mechanisms of resistance and the genetics underlying the sensitivity of cancer cells to cisplatin and other DNA damaging agents. For instance, cancers with defects in DNA damage repair pathways, such as BRCA mutations, are hypersensitive to DNA cross-linkers including cisplatin, carboplatin, and mitomycin C [85]. Other genes that have been identified as potential therapeutic targets for sensitization to cisplatin treatment include *AMBRA,* and *PRKAB1* [86, 87]*.* In fact, numerous genes have been identified as chemosensitizing targets where gene knockdown or target inhibition via small molecules sensitizes tumours to multiple chemotherapeutics. One such target is *CABYR*, a cancer testis antigen in lung cancer that sensitizes NSCLC to paclitaxel and cisplatin [88, 89]. Inhibition of components of the ATR-CHK1 checkpoint signaling pathway appears to sensitize ovarian cancer cells to multiple DNA damaging agents [86, 90]. *PAPSS1,* one target that our group identified recently, is a nuclear enzyme that produces the obligate substrate for sulfonation reactions which when inhibited, sensitizes NSCLC cells to a wide range of DNA damaging agents [91]. Swanton et al. have also identified CERT, a ceramide-binding protein, as a target that enhances the activity of cisplatin and paclitaxel in lung cancer cells, paclitaxel and 5-Fu in colorectal carcinoma cells, and paclitaxel and doxorubicin in breast cancer cells [92]. As already established through the existing principles of combination chemotherapy for treatment of cancer, maximal therapeutic effects will almost certainly involve multiple targets. This is exemplified by the studies of De et al., who demonstrated that optimal treatment outcomes were achieved in vivo when triple negative breast cancer were treated with carboplatin in combination with both an mTOR and a PARP inhibitor [93]. Similar to these studies on chemotherapeutics, groups have also aimed to identify genes that modify the sensitivity of lung cancer cells to radiation, which is commonly used in the treatment of lung cancer patients. For example, a whole genome RNAi screen found that inhibition of proteasome subunits (e.g. PSMA1) worked synergistically with ionizing radiation to induce enhanced killing of lung cancer cells [94].

#### Drug-drug interactions

Traditionally, drug combinations have been defined by simply combining two or more drugs with known single agent activity, different mechanisms of action and non-overlapping toxicities. This approach maximized potential therapeutic benefits while reducing the potential for development of resistance. These broad spectrum therapeutic approaches have led to numerous successes, but also significant failures. It is now apparent that some drug combinations work synergistically through synthetic lethality while others can result in antagonism, where the combinatorial effects turn out to be worse than what can be achieved with the single agents [96]. So the goal should be to focus on synergistic combinations; but this can be challenging as synergy can be dependent on a number of factors including drug-drug ratio, exposure time, and sequencing [95–98]. Regardless many examples of synergistic drug-drug interactions have been published and some of these have even been put into clinical practice. Peters et al. found that the combination of cisplatin and gemcitabine is synergistic in vitro and in vivo using ovarian, head and neck, and colorectal cancer models [99]. This drug combination is currently used for the treatment for at least seven different cancers types including ovarian and NSCLC. Likewise, irinotecan plus cisplatin was demonstrated to provide a synergistic benefit in the treatment of metastatic small-cell lung cancer as compared to etoposide plus cisplatin, which was the standard of care [100]. For in vitro and in vivo studies, a number of approaches have been used to assess drug-drug interactions [101, 102]. A common method uses a fixed ratio experimental designed and subsequent calculation of combination indices using the Chou-Talalay method [103, 104]. As an interesting alternative method Kang et al. analyzed over 1000 Phase II clinical trials to determined clinical synergy and antagonism of different combinations of two drugs; where overall response rates were used as the therapeutic outcome [105].

## Conclusions

### Perspectives: translating synthetic lethality for clinical applications

Although numerous synthetic lethal interactions have been discovered through genetic and chemical screening approaches, many have yet to translate into clinical successes. Translating synthetic lethal/sick discoveries to the clinic can be challenging depending on a host of factors ranging from tumor biology to the ability to access proprietary drugs own by different pharmaceutical companies. This is further complicated by the current clinical trial paradigms that typically require new treatments to be tested in patient populations that have failed standard of care therapies. An example of the latter concern is highlighted by our screen looking for cisplatin sensitizers; a screen that relies on use of lung cancer cell lines that were derived from patients that were chemo-naive. The rationale was based on the believe that optimal treatment outcomes with a combination of cisplatin and a targeted agent known to be a sensitizer would only be achieved in the first line setting. This, however, would be a clinically challenging study to complete.

**Fig. 3:**
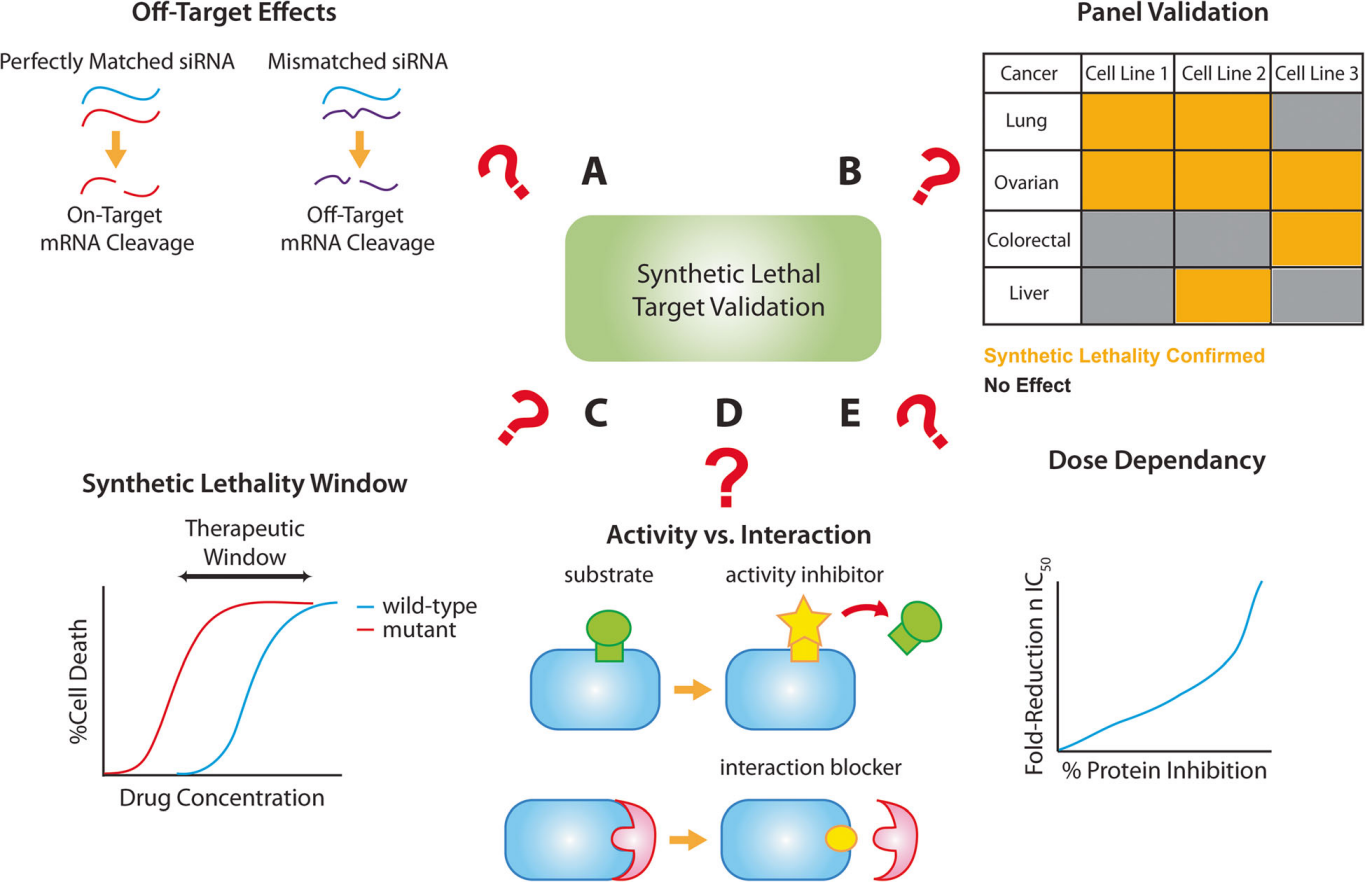
Considerations when validating synthetic lethal targets. Several factors should be considered when deciding whether or not to translate a synthetic lethal discovery to therapeutics. If the target was discovered from an RNAi screen, off-target effects should be eliminated by testing individual siRNA duplexes, using pools of siRNAs, or even testing the interaction using small molecules if available (**a**). Secondly, the synthetic lethality should be verified in a panel of cell lines for the indication(s) of interest to assess potential applications of the therapeutic strategy of interest (**b**). The therapeutic window should also be assessed to ensure that synthetic lethality occurs in a cancer-specific manner (**c**). When developing pharmaceuticals for the target of interest, it is crucial to understand whether it is the enzymatic activity or a specific interaction that is responsible for the synthetic lethality observed (**d**). Finally, synthetic lethality might be dependent on the extent of genetic alteration. This dose dependency should be explored and addressed when designing and developing therapeutics for synthetic lethal targets (**e**)

**Fig. 4:**
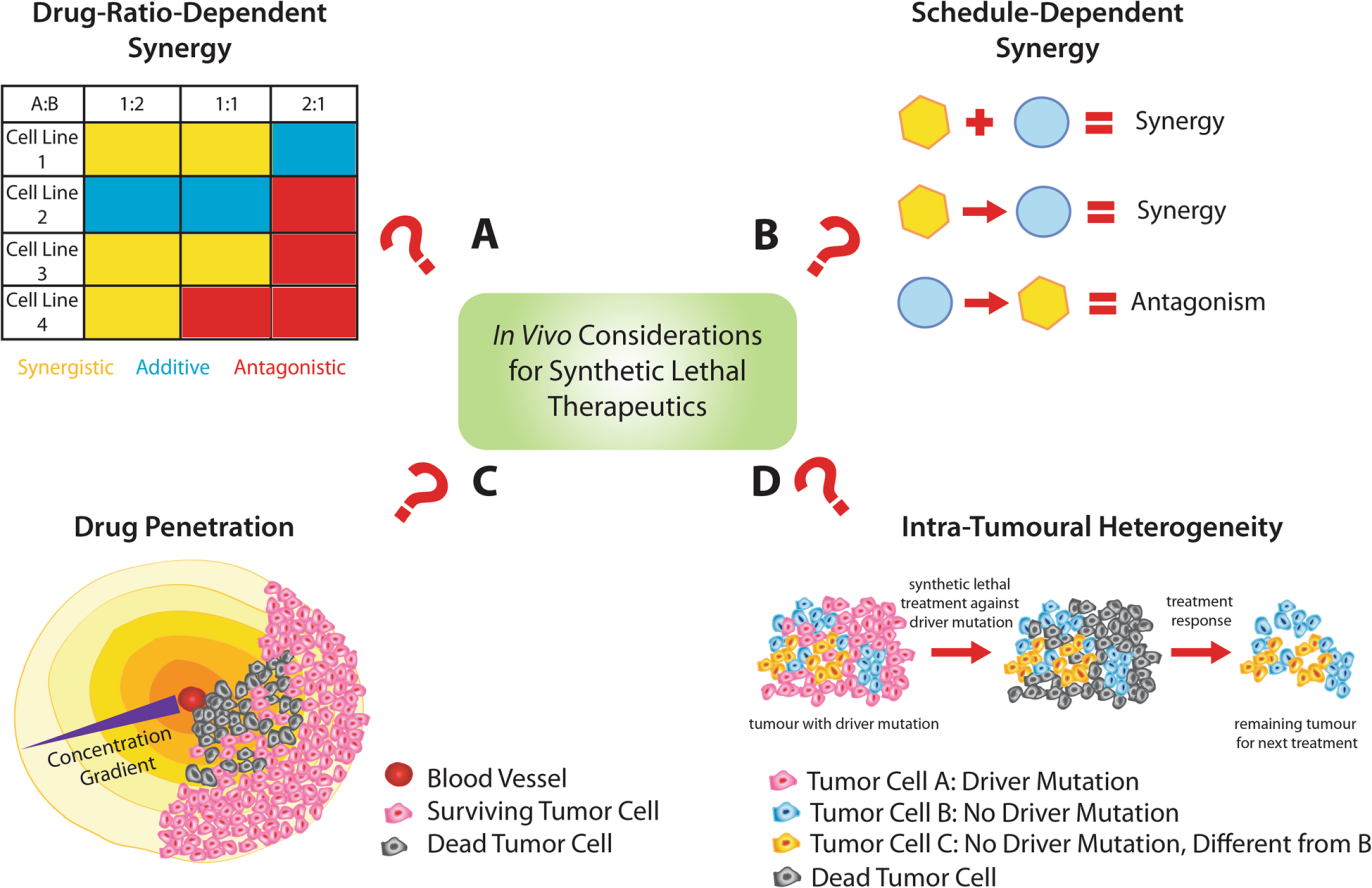
In Vivo Considerations for Synthetic Lethal Therapeutics. When using two or more therapeutics, it is important to determine the drug combination ratios at which synergy occur (**a**). This should be done in a panel of cell lines for the indication(s) of interest. Synergism may also be dependent on the timing of the administration of the different therapeutics (**b**). Another challenge that needs to be addressed is the issue associated with drug penetration into the entire tumour (**c**). As a result of poorly organized vasculature, concentration gradients will be generated upon treatment and outcomes of synthetic lethal approaches may be limited by the inability to induce sufficient genetic alterations in all cells of the targeted population. Finally, while synthetic lethal approaches are promising, certain populations of the tumour may survive treatment due to intra-tumoural heterogeneity which makes them insensitive to the specific treatment regimen (**d**)

Below, we have identified several factors that should be considered when translating synthetic lethal interactions to therapeutic strategies and have included pharmacological considerations that may be important when trying to achieve synthetic lethality interactions targeting tumours in cancer patients. These factors are summarized in Figs. 3 and 4.

### Considerations for translating synthetic lethal interactions to therapeutic strategies

When validating hits from RNAi screens, it is important to ensure that the phenotypic observations from gene-knockdown are not due to off-target effects (Fig. 3a). While mRNA sequences that perfectly match the siRNA guide strand are cleaved by the RNAi machinery, off-target silencing could also occur where the mRNA has slight mismatches with the siRNA template, particularly when the siRNA targets the 3'-UTR regions, giving rise to false positives due to unintended microRNA-like activities. These off-target effects could be minimized through strategies such as chemical modification of the siRNA duplexes and utilization of pooled sequences [106]. Validation of the on-target effects is also necessary through the use of multiple RNAi sequences to eliminate sequence-specific effects and “rescue” experiments using cDNAs. The cDNA used should lack siRNA-binding sites so that the putative target can be exogenously expressed in the presence of endogenous gene knockdown. Once the on-target effects are confirmed, it is crucial to test for synthetic lethality in a range of cell lines and relevant disease models (Fig. 3b). The goal of these validation studies is to assess the potential of synthetic lethal interaction in different contexts represented by tumour subtypes, tumours of different origin and different genetic backgrounds. It is just as important to assess the therapeutic window associated with the synthetic lethal interaction. For example, in cases where the synthetic lethal interaction is specific to an oncogenic or loss-of-function genetic background, it would be ideal that treatment causes little or no effect on cells expressing the wild-type version of the gene. The BRCA and PARP interaction highlights this point; PARP inhibitors are particularly effective against BRCA2-deficient tumours as demonstrated by Bryant et al. [42]. However, tumours harbouring wild-type BRCA2 or BRCA2-deficient tumours with BRCA2 overexpression did not respond to the treatment.

Widening of the synthetic lethality window (Fig. 3c) should also be carefully examined when validating chemo-sensitizing targets where a genetic deficiency is introduced globally to enhance the activity of another drug. Ideally, gene knockdown alone should not be deleterious to the viability of normal cells. Further, chemosensitization should be selective for only cancer cells. As an example, the target that we recently identified (PAPSS1) was found to enhance the activity of various DNA damaging agents in NSCLC cells [55] when PAPSS1 was depleted. We demonstrated that knockdown of the gene did not sensitize normal bronchial epithelial cells to cisplatin treatment while there was a > five-fold reduction in the cisplatin IC_50_ achieved when PAPSS1 was depleted in NSCLC cells.

When developing small molecules based on the findings from an RNAi screen, it is important to understand the specific function of the gene product (Fig. 3d) involved in the synthetic lethal interaction. Since transcription factors and other non-enzyme targets are generally considered “undruggable”, candidates with enzymatic activities such as kinases are prioritized for target development with the assumption that the enzymatic activity is responsible for the synthetic lethality observed in an RNAi screen. However, it is a possibility that the effect is the result of interactions between proteins rather than enzymatic function. In 2009, Scholl et al. discovered from an RNAi screen that *KRAS*-driven cancers are dependent on a gene that encodes for a serine/threonine protein kinase STK33 [58]. In 2012, Luo et al. developed a potent and selective kinase inhibitor for STK33 which failed to reproduce the synthetic lethality observed in the original screen [107]. It can be argued that this is due to the fact that STK33 has other non-kinase functions that are critical to the viability of *KRAS*-driven cancer cells, for example protein scaffolding. Such protein-protein interactions could be explored through co-immunoprecipitation studies or through more sophisticated approaches such as tandem affinity purification during target validation [108]. While small molecule inhibitors may be developed to inhibit the enzymatic activity of a candidate target, peptides or peptide mimetics could be developed to inhibit specific protein-protein interactions important for synthetic lethality [109]. This rather novel therapeutic area also opens up opportunities for targeting the traditionally “undruggable” hits from synthetic lethal screens.

It should be noted that synthetic lethal targets discovered in yeast studies are based on complete gene knockouts, therefore these interaction are “definitive.” In contrast, RNAi screens utilizing siRNA or shRNA rarely eliminate the gene product completely and therefore the amount of depletion achieved may be critical to defining the interaction. For this reason, it is important to determine the minimal level of protein depletion necessary to achieve the desired synthetic lethal/sick effect. For instance, our studies on PAPSS1 showed that sensitization to cisplatin treatment occurred in a siRNA dose-dependent manner [91]. At the protein level, at least 80 % inhibition relative to scramble controls was necessary to achieve a meaningful improvement in cisplatin activity. While the extent to which the protein activity/interaction is inhibited can be determined in vitro and perhaps adjusted through a medicinal chemistry campaign. This dose dependency (Fig. 3e) will, however, be more challenging to address in vivo, as discussed in the following section.

### Optimizing multidrug combinations to induce synthetic lethality

As indicated previously, the success of multidrug combinations is largely dependent on ensuring, in vivo, that the combinatorial effect is maintained at the site where the cancer resides. The factors influencing synergistic or synthetic sick interactions can be better understood through carefully designed in vitro studies that consider drug concentrations, exposure time, sequence and dosing parameters (e.g. drug/drug ratio). However, in the context of achieving a synthetic lethal interaction in vivo, one must have an excellent understanding of the drug pharmacokinetics and biodistribution attributes. Traditionally, drug combinations were given at maximally tolerated doses to achieve the greatest therapeutic effects. However, studies in the last decade have found that drug combinations display drug-ratio-dependent synergy (Fig. 4a) [110]. For instance, the combination of cisplatin and irinotecan, which is an approved combination for the treatment of lung cancer, was screened by Tardi et al. in a panel of 20 cell lines over a range of drug ratios [111]. Their study indicated that an antagonistic region (irinotecan/cisplatin molar ratios 1:2 to 4:1) was consistently detected in these cell lines. Importantly, the regions where synergy was observed (<1:2 and >4:1) were conserved in vivo. These results raise an important consideration that some drug combinations may need to be administered in a manner that can maintain an optimal synergistic ratio. This, however, can be challenging as different drugs exhibit different adsorption, distribution, and metabolism profiles. One approach to address this has been through the use of drug delivery technology. By co-encapsulating the two drugs at the optimally synergistic ratio into nanocarriers such as liposomes, the pharmacokinetic profile of both drugs can be controlled to maintain the drug ratio in vivo [110]*.* As an example, VYXEOS (CPX-351) is a liposomal formulation comprising cytarabine and daunorubicin (5:1 molar ratio) that was developed using the Combiplex® technology for the treatment of acute myeloid leukemia (AML) [97, 112, 96]. The Phase III clinical trial with VYXEOS has recently been completed and the results indicated that this formulation comprising two drugs was more effective than the standard of care 7 + 3 cytarabine/daunorubicin treatment [113].

While drug-drug ratios can be optimized when drug combinations are given concurrently, if the drug interactions are schedule-dependent (Fig. 4b), then one may have to optimize how treatments are sequenced. For instance, in a study conducted by Li et al.*,* combinations of pemetrexed and erlotinib where synergistic when the two drugs concurrently and also when permetrexed was administered first followed by erlotinib [114]. However, the same two drug combination was antagonistic when erlotinib was given before pemetrexed. In another previous study, the use of the liposomal irinotecan formulation Irinophore C™ in combination with 5-FU concurrently resulted in high levels of toxicity. However this toxicity was reduced substantially when 5-FU was administered sequentially following Irinophore C™ in colorectal cancer models [115, 97]. Similarly, a phase III node-positive breast cancer trial demonstrated significantly better overall survival when patients were given doxorubicin and docetaxel sequentially relative to concurrent chemotherapy [116]. Although these examples represent classic drug-drug combinations, it is very likely that combinations arising from synthetic lethal/sick screens will be influenced by the similar factors.

Finally, when translating synthetic lethality to therapeutic strategies, it is imperative to consider intra- and inter-tumoural heterogeneity as well as the tumour microenvironment. The tumour microenvironment has long been known to significantly limit drug penetration (Fig. 4c). Tumor cells are exposed to sub-lethal doses of drug and this contributes to treatment failures [117]. Due to the poorly organized vasculature in tumours, drug treatment with small molecules create concentration gradients that lead to reduced drug exposure at certain regions of the tumour. While screening strategies have been used to identify synthetic lethal gene partners that can be inhibited to enhance the cytotoxic effects of low-dose chemotherapeutics, these therapeutic strategies may again be limited by the same drug penetration issues, the level of hypoxia, as well as the nutritional status of the target cells. If, as indicated earlier, a minimal level of target inhibition/depletion is needed to achieve a desired synthetic lethal or synthetic sick effect, then this must be achieved throughout the tumour. Further it will be important to validate that the interaction still occurs under conditions where cells may be stressed due to lack of oxygen or nutrients. In the context of the target identified in our studies, PAPSS1, a significant challenge associated with the future development of gene, peptide, or small molecule therapy against this target would be the need to achieve 80 % PAPSS1 knockdown in all regions of tumours that are exposed to low-dose cisplatin. Although there will always be a drug concentration gradient, the effects could potentially be mitigated through the use of drug delivery systems. As an example, liposomal formulations of doxorubicin, which are clinically approved, have shown to be efficacious through increased circulation lifetime leading to increased drug accumulation at the tumour site [118, 120]. Although concentration gradients would still be generated in that case, a greater amount of drug would be available even at hypoxic or nutrient-deprived regions due to greater overall exposure to the therapeutic agent.

In terms of future perspectives, synthetic lethality is a promising approach at the cellular level and even at the population level if the tumour population is clonal. In reality, however, intra-tumoural heterogeneity (Fig. 4d) contributes tremendously to the challenge of curing cancer. By use of chemotherapy and/or targeted agents, one treatment regimen could potentially eradicate a large population of tumour cells. At the same time, this treatment may remove selective pressures against existing dormant tumour cells. Even in the context of tumours with driver mutations, which could potentially be targeted using synthetic lethal strategies, not all tumour cells would harbour the specific driver mutation simply due to the countless mutations acquired through numerous generations. Nonetheless, we believe that treatments arising out of the principles of synthetic lethality, if applied early, should extend patient survival. With the use of other advanced technologies, such as post-treatment sequencing of tumour biopsies, continuous applications of synthetic lethal strategies will allow the disease to be managed for a longer period of time, ultimately making aggressive and difficult to treat cancers, such as lung cancer, a chronic disease instead of a terminal, illness.

## Abbreviations

AML: Acute myeloid leukemia
DDR: DNA damage response
GBM: Glioblastoma multiforme
GOF: Gain-of-function
HR: Homologous recombination
LAC: Lung adenocarcinoma
LOF: Loss-of-function
NHEJ: Non-homologous end joining
NSCLC: Non-small cell lung cancer
SCLC: Small cell lung cancer
SqCC: Squamous cell carcinoma
TKI: Tyrosine kinase inhibitors
